# Redox Molecules in Aging and Neurodegenerative Disorders

**DOI:** 10.3390/ijms27156567

**Published:** 2026-07-23

**Authors:** Igor Y. Iskusnykh, Evgenii D. Kryl’skii, Tatyana N. Popova, Anastasia A. Zakharova

**Affiliations:** 1 Department of Anatomy and Neurobiology, University of Tennessee Health Science Center, Memphis, TN 38163, USA; 2 Department of Medical Biochemistry, Molecular and Cell Biology, Voronezh State University, Universitetskaya Sq. 1, Voronezh 394018, Russia; 3 Department of Medical Biochemistry, Faculty of Biomedicine, Pirogov Russian National Research Medical University, Ostrovitianov St. 1, Moscow 117997, Russia

**Keywords:** oxidative stress, Alzheimer’s disease, neuron, ROS, antioxidant, neurodegeneration, Parkinson’s disease, brain, mitochondria, glutathione

## Abstract

Oxidative stress is a key player in the pathogenesis of aging and various neurodegenerative disorders, including Alzheimer’s disease (AD), Parkinson’s disease (PD), Huntington’s disease (HD), and multiple sclerosis (MS), among others. Coupled molecules involved in chemical reduction-oxidation (redox) reactions regulate central signaling pathways and ensure the activation of cytoprotective mechanisms as needed. Such redox couples include oxidized and reduced forms of nicotinamide adenine dinucleotide (NAD^+^/NADH), nicotinamide adenine dinucleotide phosphate (NADP^+^/NADPH), and glutathione (GSSG/GSH), respectively. Under pathological conditions, concentrations of NAD^+^, NADPH, and GSH decrease, and an imbalance between the oxidized and reduced forms of these molecules develops. The current review focuses on the mechanisms that underlie these changes and their potential consequences for neurodegenerative processes and aging. The review also evaluates experimental studies on metabolic and genetic abnormalities associated with alterations in NAD^+^, NADPH, and GSH concentrations and highlights current research on strategies to regulate these compounds for neuroprotective purposes. Special attention is paid to understanding the interconnection between disturbed redox homeostasis and mitochondrial dysfunction, as this crosstalk is increasingly recognized as a crucial step in neurodegeneration. Integration of biochemical, genetic, and therapeutic perspectives provides a comprehensive understanding of redox imbalance in the pathogenesis of aging and neurodegenerative disorders. Therefore, these insights may contribute to the development of innovative interventions that target redox homeostasis, with the potential of increasing the human lifespan.

## 1. Introduction

The redox couples of NAD^+^/NADH and NADP^+^/NADPH and the thiol/disulfide system involving reduced glutathione (GSH) and its oxidized form (GSSG) play critical roles in regulating redox homeostasis. The NAD^+^/NADH ratio is a key determinant of cellular redox status and plays a central role in regulating catabolic metabolism and energy production. A delicate equilibrium is maintained between the concentrations of free NADH and NAD^+^ on one hand and the levels of reduced and oxidized substrates for essential cellular dehydrogenases on the other hand. NADPH and NADP^+^ are essential for anabolism, antioxidant defense mechanisms, and regulation of the thiol/disulfide balance, thereby contributing to the overall redox status of the organism. The NADH/NAD^+^ and NADPH/NADP^+^ redox systems are interconnected through mitochondrial nicotinamide nucleotide transhydrogenase, which links these redox couples and contributes to their coordinated regulation. NADPH serves as a reducing agent during anabolic processes, such as the synthesis of fatty acids and amino acids [1].

The formation of NADH from NAD^+^ occurs mainly during glycolysis, the TCA cycle, and fatty acid (FA) oxidation. NADH supplies electrons to the electron transport chain, thereby producing ATP while being oxidized back to NAD^+^. NAD^+^ also serves as a substrate for various enzymes, including NAD^+^-dependent deacetylases (Sirtuins or SIRTs), Poly (ADP-ribose) polymerases (PARPs), NAD^+^ glycohydrolase, and cyclic ADP ribose (cADPR) synthase. These enzymes are involved in physiological and biochemical processes that include energy metabolism, DNA repair, regulation of gene transcription, epigenetic genome regulation, and cellular aging [2]. Moreover, NAD^+^ can be converted to NADP^+^ by NAD^+^ kinases. The redox ratio of NAD^+^/NADH is a key regulator of cellular energy metabolism that influences the activity of glycolytic enzymes, the TCA cycle, oxidative phosphorylation (OXPHOS) in mitochondria, and other processes [3].

The NADPH-dependent thioredoxin (TRX) system operates in a similar manner using NADPH, while NADPH binding helps maintain catalase stability and activity. Low-molecular-weight thiol compounds, of which GSH is the most abundant, are ubiquitous in living organisms, where they serve as redox buffering agents that shield cells from the harmful effects of reactive molecules. The conversion of GSSG to GSH is catalyzed by glutathione reductase (GSR), which reduces GSSG to the potent antioxidant GSH. Glutathione serves as a substrate for other antioxidant enzymes, including glutathione-S-transferase (GST) and glutathione peroxidases (GPx) [4,5]. GSH can interact directly with oxidants and cysteine residues in proteins via the reversible process of S-glutathione conjugation. GSH also plays a crucial role in the recycling of ascorbate and provides reducing equivalents for the enzymes glutathione peroxidase (GPx) and peroxiredoxin (PRx) [4,6]. Methionine residues in proteins can also undergo reversible oxidative modification by their conversion to methionine sulfoxide, which can be reduced by methionine sulfoxide reductase enzymes [7].

Thus, the purpose of this review is to outline the role of redox molecules such as NADH, NADPH, and GSH in the development of neurodegenerative diseases and aging. It is anticipated that these insights may contribute to the development of innovative interventions for both fundamental research and clinical applications.

## 2. From Metabolism to Maladaptation: NAD^+^ Decline as a Driver of Aging Disorders

Normal and pathological aging are accompanied by a metabolic betrayal. NAD^+^ is abundant in youth and essential for cellular vitality, but its level gradually declines with time. This section reveals how its decline rewires metabolism, leading to maladaptation and the rise of age-related disorders.

### 2.1. Molecular Pathways of NAD^+^ Biosynthesis

NAD^+^ is synthesized via three main pathways: de novo biosynthesis, the Preiss-Handler pathway, and the salvage pathway. In mammals, the kynurenine pathway is the major de novo pathway for NAD^+^ biosynthesis. It begins with the catabolism of the amino acid tryptophan, generating kynurenine as a key intermediate that can be further metabolized toward NAD^+^ production or diverted to the formation of kynurenic acid and xanthurenic acid. The kynurenine pathway regulates neuronal function by contributing to the synthesis of two major neurotransmitters (glutamate and acetylcholine), regulating the activity of N-methyl-D-aspartate receptors, and producing free radicals. This pathway produces not only neuroprotective compounds, such as kynurenic acid, but also some neurotoxic metabolites such as 3-hydroxykynurenine, 3-hydroxyanthranilic acid, and quinolinic acid [8].

It has been demonstrated that kynurenine aminotransferase II, an enzyme that converts kynurenine into kynurenic acid, is strongly localized to astrocytes. Kynurenine aminotransferase II (KAT II/AADAT), an enzyme responsible for kynurenic acid synthesis, is expressed in the brain, including regions involved in adult neurogenesis such as the hippocampus and subventricular zone [9].

There is evidence of alterations in the activity of certain enzymes in the kynurenine pathway in the brain as a function of age. Specifically, there is an inhibition of TRP-2,3-dioxygenase and an increase in the activity of indoleamine-2,3-dioxygenase [10].

Elevated levels of quinolinic acid, 3-hydroxy-L-kynurenine, and 3-hydroxyanthranilic acid oxygenase have been reported in the brains of patients with early-stage Huntington’s disease (HD), as well as a decrease in the activity of kynurenine aminotransferase. Additionally, a significant increase in kynurenine-3-monooxygenase activity and a decrease in kynurenase activity were observed in a transgenic mouse model of HD. Increased regulation of indolamine-2,3-dioxygenase 1 and production of quinolinic acid have also been linked to Alzheimer’s disease (AD), moreover, elevated concentrations of kynurenic acid were found in the shell and caudate nucleus of patients with AD. Reduced levels of kynurenic acid and increased levels of 3-hydroxy-L-kynurenine were found in the brains of individuals with Parkinson’s disease (PD) [11].

In the Preiss-Handler and salvage pathways, NAD^+^ is produced from pyridine derivatives. The Preiss-Handler pathway generates NAD^+^ from nicotinic acid (NA) in a three-step process via the intermediate nicotinic acid adenine dinucleotide (NAAD). A key component of the Preiss-Handler pathway is nicotinamide mononucleotide adenylyltransferase (NMNAT). The salvage pathway, also known as the NAD^+^ bypass route, begins with the conversion of nicotinamide (NAM) to nicotinamide mononucleotide (NMN) via the intracellular nicotinamide phosphoribosyltransferase (NAMPT), followed by the conversion of NMN to NAD^+^ via NMNAT. The salvage pathway also involves the conversion of nicotinamide riboside (NR) to NMN via nicotinamide riboside kinase 1 or 2 (NRK1/NRK2) [8]. At present, there is limited information available regarding how aging impacts NAD^+^ biosynthesis within the Preiss-Handler pathway. The decrease in NAD^+^ synthesis with aging can also be attributed to a reduction in the levels of NMNAT isoforms, as observed in oocytes of elderly mice. Furthermore, increased NMNAT expression in Drosophila has been shown to extend the lifespan by enhancing their response to oxidative stress and improving mitochondrial function [12]. In typical Wallerian degeneration resulting from trauma, NMNAT2 expression is lost, accompanied by an increase in the NMN/NAD ratio, which contributes to possible axonal degeneration [13]. There is evidence to suggest that NMNAT2 may have a chaperone function, protecting neurons in the cerebral cortex from protein-induced stress. Additionally, the enzymatic activity of NMNAT2 appears to protect against excitotoxic damage. NMNAT2 levels have been found to be decreased in the brains of individuals with asthma, whereas NMNAT1 levels remain unchanged [14]. In Tg2576 mice, a model of Alzheimer’s disease pathology, reduced NMNAT2 levels have been observed in the cerebral cortex and hippocampus and were associated with increased tau phosphorylation [15]. In ICR mice, it has been shown that the level of NAMPT in the brain decreases with age, particularly in the hippocampus and cerebellum, and increases in serum. In the brain, NAMPT, as an enzyme, is involved in NAD^+^ biosynthesis and promotes cell survival. Extracellular NAMPT may function both as an enzyme and as a pro-inflammatory cytokine. The levels of NAMPT change in opposite directions in different cell types in the brain during aging, increasing in microglial cells and decreasing in neuronal cells. NAMPT is not present in the microglia of young mice but is highly expressed in elderly mice, particularly in the hippocampus and cerebellum. These changes may contribute to microglia-mediated neuroinflammation in aging [16].

### 2.2. NAD^+^-Dependent Metabolic and Signal Pathways

NAD^+^ is a critical redox factor in metabolic processes and ATP synthesis. Beyond its role in cellular bioenergetics, NAD^+^ acts as a substrate for a variety of enzymes that catalyze the breakdown of NAD^+^ to NAM. These enzymes include class III histone deacetylases (SIRTs), poly(ADP-ribose) polymerases (PARPs), ADP-ribosyl cyclases (CD38/CD157), and the NAD^+^ hydrolase sterile alpha and TIR motif containing 1 (SARM1), will be described in more detail below [8].

SIRT enzymes are sensitive to intracellular concentrations of NAD^+^ and relay signal transmission through the deacetylation of various proteins. Mammals have seven SIRTs in different subcellular compartments, including nuclear SIRT1, SIRT6, and SIRT7, cytoplasmic SIRT2, and mitochondrial SIRT3, SIRT4, and SIRT5. SIRT1 functions as an epigenetic regulator by deacetylating specific acetylated histone residues, such as H3K9, H3K14, and H4K16. SIRT1 also affects transcription by deacetylating specific transcription factors, such as TP53, NF-κB, PGC-1α, and FOXO3a. SIRT1 activity is influenced by NAD^+^ availability, which is regulated in part by NAMPT-mediated NAD^+^ biosynthesis through the salvage pathway [17,18]. Mitochondrial SIRT3 inhibits axon degeneration, regulates ATP production via deacetylation of respiratory chain complex I protein, and modulates opening of the mitochondrial permeability transition pore (mPTP) via regulation of cyclophilin D activity [19].

It has been demonstrated that the levels of SIRT3-6 decrease with age in some areas of rat brain. At the same time, the level of SIRT7 increases in the frontal cortex. The SIRT2 level increases with aging in a region-specific manner. In the brains of aged rats, there is also a decrease in SIRT1 level or activity in the hippocampus [20].

PARP-associated proteins are encoded in humans by 17 genes. Of these, the most widely expressed is PARP1, which is activated in response to DNA damage and plays a role in DNA repair and the regulation of programmed cell death (apoptosis). Upon activation, PARP1 and PARP2 catalyze the addition of multiple ADP-ribose moieties from NAD^+^ onto acceptor proteins, resulting in the formation of long chains of poly(ADP-ribose) (PAR). This process leads to the depletion of significant amounts of cellular NAD^+^, a crucial coenzyme that is involved in numerous cellular processes [17].

PARP1 has been suggested to play a role in the regulation of aging as an antagonistic pleiotropic molecule. On the one hand, it provides protection to cells from aging in physiological conditions. On the other hand, it can promote cell death or decreased functionality in older individuals or in pathophysiological conditions. Excessive PARP1 activation due to DNA damage and chronic neuroinflammation may accelerate aging in mice with the A-T mutation. Conversely, efficient PARP1-mediated DNA repair has been associated with longevity in some human studies, although the relationship between PARP1 activity and lifespan remains complex [21].

It was demonstrated that in rats at the age of 14 months, PARP activity increased in nuclear fractions of the hippocampus, cerebellum and cerebral cortex compared to younger individuals. In animals aged 24–27 months, PARP activity in the hippocampus was approximately 50% lower, while it remained unchanged in the cerebral cortex and cerebellum compared to rats aged 14 months. Reduced enzyme activity in the hippocampus may impair its ability to repair damaged DNA, potentially increasing the vulnerability of hippocampal neurons to various toxins [22]. Oxidative stress induces DNA damage and depletes NAD^+^ through PARP-mediated poly(ADP-ribosyl)ation [23]. However, ROS are not only harmful byproducts but also important signaling molecules that regulate gene expression. For example, estrogen-induced LSD1-dependent histone demethylation generates transient ROS and oxidative DNA lesions that contribute to gene expression changes, highlighting a link between redox signaling and chromatin remodeling. In contrast, sustained ROS accumulation can shift the cellular response from adaptive signaling toward oxidative damage and apoptosis [24]. Moreover, excessive ROS can activate ATM/p53 signaling, leading to mitochondrial dysfunction and apoptosis [25,26]. Interestingly, age-related declines in androgen levels may increase susceptibility to neurodegeneration. Testosterone and dihydrotestosterone promote neuronal survival through androgen receptor-dependent activation of the MAPK/ERK pathway, leading to activation of p90 ribosomal S6 kinase (RSK) and phosphorylation-mediated inactivation of the pro-apoptotic protein Bad [27].

Another group of NAD^+^ consumers is the ADP ribosyl cyclases that use NAD^+^ as a substrate to produce cyclic adenosine diphosphate ribose (cADPR), an important intracellular regulator of Ca^2+^ homeostasis. This cADPR synthase activity indirectly affects many Ca^2+^-regulated processes, including cellular proliferation, muscle contraction, immune responses, and secretion of glucose-induced insulin from pancreatic beta cells. Two well-established members of this family are the homologues CD38 and CD157, thought to have resulted from a gene duplication [8,28]. Age-associated increases in CD38 expression and activity have been proposed to contribute to NAD^+^ depletion during aging. Reduced NAD^+^ availability may impair mitochondrial function by limiting NAD^+^-dependent processes, including sirtuin-mediated regulation of mitochondrial metabolism [8]. CD38 expression and enzymatic activity increase with age in the brains of rats with spontaneous hypertension and that are prone to stroke, and are associated with oxidative stress [29]. At the same time, no studies have directly shown an increased CD38 expression in the human brain due to aging, or increased levels of CD38 in the brains of patients with neurodegenerative diseases compared to an age-matched control group. Nevertheless, aging is a major risk factor for the majority of neurodegenerative disorders, and some evidence suggests that CD38 expression may increase in the brain with aging [30].

A significant consumer of NAD^+^ in neuronal cells is the NAD^+^ hydrolase SARM1. SARM1 contains a Toll/Interleukin (IL)-1 receptor (TIR) domain that forms homodimers, a process that converts NAD^+^ to NAM and ADP-ribose and produces a small quantity of cADPR [8]. Thus, small molecule analogues of NAD^+^ may act as SARM1 inhibitors. SARM1 is a central player in programmed axonal degeneration, which can be triggered by necroptosis, genetic disorders, toxic metabolic disturbances, physical injury, and neuroinflammation [31].

**Figure 1:**
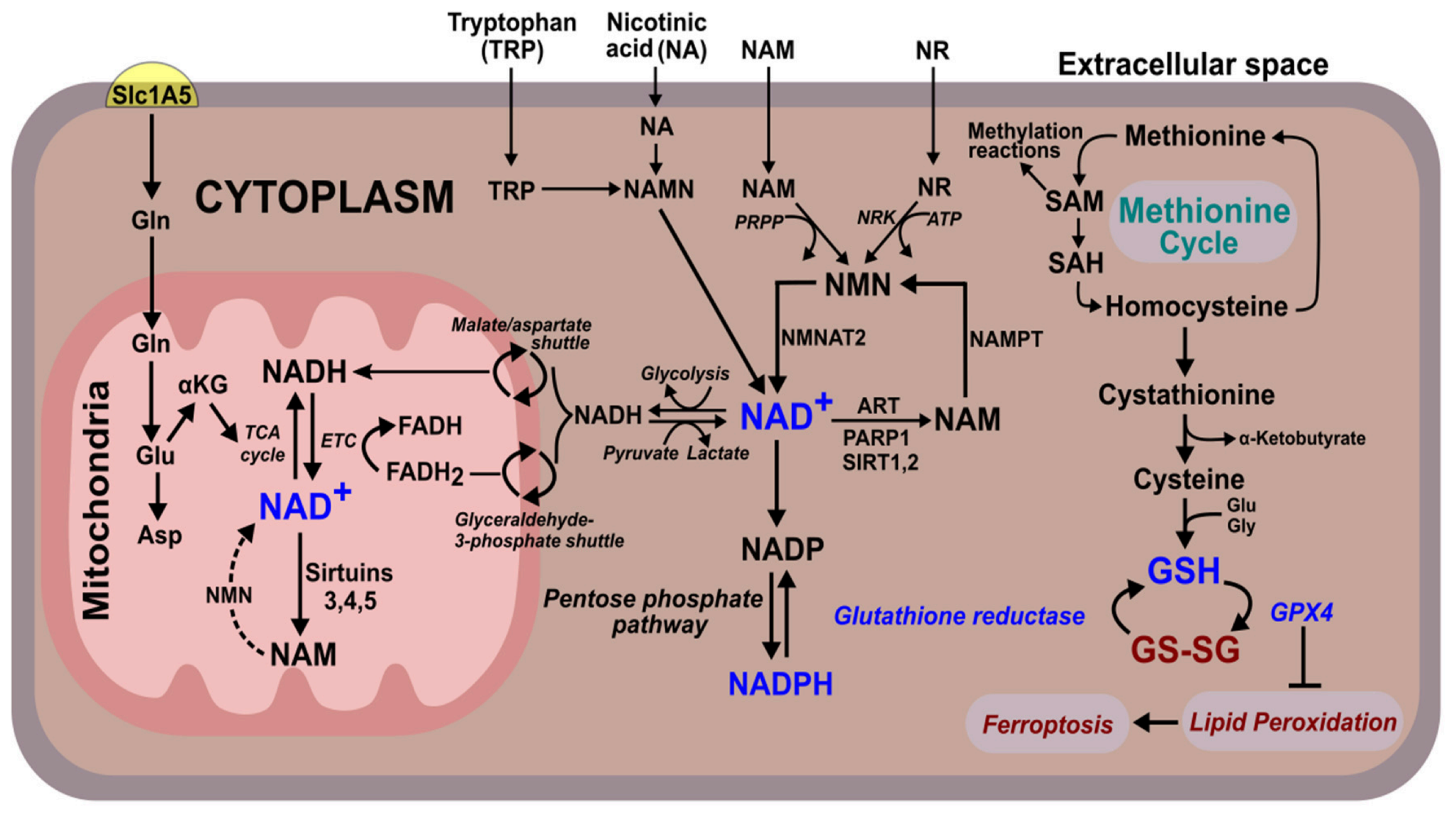
Overview of NAD^+^ Metabolism and Its Interconnections with NADP(H), Glutathione, and Ferroptosis. Modified from Verdin, Science, NAD^+^ in aging, metabolism, and neurodegeneration, DOI: 10.1126/science.aac4854 [2015], AAAS [17].

Although several classes of proteins utilize NAD^+^, they play different roles in the process of aging and degeneration. Sirtuins are generally associated with pro-longevity pathways, whereas excessive activation of NAD^+^-consuming enzymes such as PARPs, CD38, and SARM1 can contribute to NAD^+^ depletion and age-related dysfunction. SIRT1, PARP1, and CD38 consume NAD^+^ and may compete for limited intracellular NAD^+^ pools. Age-related accumulation of DNA damage increases PARP1 activation and NAD^+^ consumption, which may reduce NAD^+^ availability for SIRT1-dependent signaling pathways [17,32]. NAD^+^ metabolism comprises multiple interconnected enzymatic steps and is closely linked to NADP(H) metabolism and glutathione metabolism, requiring tight regulation to maintain cellular redox homeostasis [17,33,34,35,36,37], with selected aspects of these processes illustrated in Figure 1.

This figure illustrates the interplay between NAD^+^ metabolism, glutathione (GSH) biosynthesis, and redox pathways. It also represents the major intracellular NAD^+^ precursors, including tryptophan (Trp), nicotinic acid (NA), nicotinamide, nicotinamide riboside (NR), and nicotinamide mononucleotide (NMN). The mitochondrial membrane is impermeable to NAD^+^ and NADH. Reducing equivalents produced during glycolysis are transferred into the mitochondrial matrix through the malate–aspartate and the glycerol-3-phosphate shuttles. Mitochondrial NADH generated via the malate-aspartate shuttle is oxidized by complex I of the electron transport chain (ETC), whereas FADH_2_ produced via the glycerol-3-phosphate shuttle is oxidized by complex II. NAD^+^-consuming enzymes generate nicotinamide, which is recycled through the NAD^+^ salvage pathway. The metabolism of glutamine (Gln), glutamate (Glu), and α-ketoglutarate (αKG) is closely integrated with cellular energy metabolism. Glutamine is converted to glutamate and subsequently to αKG, which enters the tricarboxylic acid (TCA) cycle to support ATP production. NADPH, generated in the pentose phosphate pathway, maintains the antioxidant activity of glutathione reductase. The methionine cycle generates S-adenosylmethionine (SAM) and S-adenosylhomocysteine (SAH), where SAM serves as the primary methyl donor for DNA and protein methylation reactions; SAH is converted to homocysteine, which is subsequently metabolized to support glutathione (GSH) synthesis. Glutathione reductase (GR) uses NADPH to reduce oxidized glutathione (GSSG) back to reduced glutathione (GSH), thereby maintaining the intracellular GSH pool and cellular redox homeostasis. GSH serves as an essential reducing substrate for glutathione-dependent GPXs, including glutathione peroxidase 4 (GPX4), enabling the detoxification of lipid peroxides and preventing lipid peroxidation and ferroptosis.

### 2.3. The Central Role of NAD^+^ in Aging and Metabolic Homeostasis

During the process of normal aging, the levels of NAD^+^ in various tissues are reduced, perhaps due to an increase in NAD^+^ consumption by PARPs and CD38, as noted above, and a decrease in NAD^+^ synthesis and NAMPT activity [8]. Such a reduction is observed in multiple species. In C57BL/6J mice, a reduction in the levels of NAD^+^ in the nucleus is associated with decreased SIRT1 activity and impaired regulation of mitochondrial transcription factor A (TFAM), thereby linking NAD^+^ loss to mitochondrial dysfunction. Interestingly, a reduction of nuclear NAD^+^ levels during aging is associated with a reduction in SIRT1 activity and decreased hippocampal expression of TFAM, which is linked to the development of asthma in human patients and in an experimental model in mice. Conversely, an increase in TFAM expression normalizes the activity of the electron transport chain complex 1 and improves the cognitive abilities of older mice. A loss of SIRT3 activity in the muscles of aged rats is linked to the induction of a pseudo-hypoxic state, similar to that seen in aging, after disruption of the electron transport chain [38,39]. CD38 is an NAD^+^-consuming enzyme involved in immune cell regulation. Activation of CD38 can reduce cellular NAD^+^ levels through NAD^+^ hydrolysis, linking CD38 activity to regulation of cellular metabolism and immune signaling [40].

During ischemia/reperfusion injury, PARP1 activation contributes to neuronal cell death through mitochondrial dysfunction and apoptosis-inducing factor (AIF) translocation. In rat brain and HT22 hippocampal neuronal cells, inhibition of PARP1 activation and AIF translocation reduces oxidative stress-induced cell death [41]. Similar events have also been observed in the cortex and hippocampus of rats with Alzheimer’s disease (AD) [42]. Human neurons deficient in autophagy components exhibit reduced NAD^+^ levels due to increased NAD^+^ consumption, leading to mitochondrial dysfunction and neuronal cell death [43].

### 2.4. NAD^+^ at the Crossroads of Metabolic Dysfunction and Neurodegeneration

NAD^+^ depletion is particularly detrimental to the nervous system and is commonly observed in various neurodegenerative disorders. As noted above, overexpression of the enzyme CD38 leads to NAD^+^ deprivation, which may indirectly contribute to neuroinflammation. As individuals age, NAD^+^ levels naturally decrease due to the activation of PARPs, which is associated with an increase in oxidative DNA damage, such as that seen in Parkinson’s disease (PD) and other brain disorders [44]. NAD^+^ depletion has also been observed in diseases that are associated with accelerated aging, including ataxia-telangiectasia (AT), xeroderma pigmentosum group A (XPA), and Cockayne syndrome (CS) [8].

The most well-known neurodegenerative disorders include AD, PD, and amyotrophic lateral sclerosis (ALS), which share the characteristic of axon loss prior to neuronal cell death. In the context of Wallerian degeneration, a decrease in the activity of NMNAT2 results in an increased ratio of NMN to NAD^+^ that, in turn, activates the NADase activity of SARM1. This process contributes to further NAD^+^ depletion and axon degeneration. Interestingly, genetic knockout (KO) of the SARM1 gene has a significant neuroprotective effect on some of these disorders [13,45,46]. The accumulation of DNA damage in Parkinson’s and Alzheimer’s diseases contributes to PARP1 hyperactivation, resulting in rapid NAD^+^ consumption and impaired cellular energy metabolism. In experimental models, genetic or pharmacological inhibition of PARP1 has been shown to improve mitochondrial function, reduce neuronal damage, and ameliorate disease-associated phenotypes [13,47].

A decrease in the intracellular concentration of NAD^+^ as a result of pathological conditions disrupts the functioning of SIRT proteins, particularly SIRT1. It was demonstrated that the addition of the tau K18 protein (fragment of full-length human tau protein containing four microtubule-binding repeats domain) to the BV2 microglial cell line resulted in a significant reduction in SIRT1 expression [38]. Also, in another study, NAD^+^ deficiency reduces SIRT1 activity, impairing mitochondrial regulation and contributing to electron transport chain dysfunction and the development of a pseudo-hypoxic state in skeletal muscle during aging [39].

Reduced nuclear NAD^+^ availability during aging and disease decreases SIRT1 activity, impairing PGC-1α activation and contributing to mitochondrial dysfunction and reduced mitochondrial biogenesis. Altered NAD^+^ metabolism has also been associated with mitochondrial abnormalities and impaired SIRT1 signaling in Alzheimer’s disease. NAD^+^ depletion may compromise mitochondrial quality control mechanisms, including mitophagy, through reduced activity of NAD^+^-dependent sirtuins involved in autophagy regulation and mitochondrial maintenance. Furthermore, adequate NAD^+^ availability is required for DNA repair pathways mediated by PARPs and NAD^+^-dependent sirtuins such as SIRT1 and SIRT6. Interestingly, NAD^+^ availability influences neuronal function through NAD^+^-dependent pathways, including SIRT1 signaling, which may regulate neuroprotective mechanisms such as brain-derived neurotrophic factor (BDNF) expression. BDNF plays an essential role in neuronal survival, stress adaptation, and synaptic plasticity in the central and peripheral nervous systems. Through activation of TrkB-dependent signaling pathways, BDNF contributes to glucose metabolism and mitochondrial biogenesis, supporting neuronal function during aging [8,13].

Thus, NAD^+^ depletion is linked to neurodegeneration via a number of pathways that affect the energy supply to neurons and the function of protective mechanisms within cells in the central nervous system. A summary of research on NAD^+^ levels during aging and neurodegenerative conditions is presented in Table 1.

### 2.5. Strategies for Restoring NAD^+^ Levels

Various methods that increase NAD^+^ levels have the potential to delay the onset of aging in diseases that are associated with premature aging. Calorie restriction and conditions that induce energy stress, such as physical activity or decreased glucose availability, contribute to increased NAD^+^ levels, leading to increased SIRT activity, which regulates transcription of genes involved in oxidative metabolism, antioxidant system function, and DNA repair processes [63]. An increase in the level of NAD^+^ may also enhance mitochondrial function. In mouse cardiomyocytes, the mitochondrial protein SIRT3 plays a crucial role in the protective effects of NAD^+^-dependent mechanisms by preventing apoptosis and inhibiting the opening of the mitochondrial permeability transition pore (mPTP) with age. SIRT3 also promotes nuclear translocation of the forkhead box factor O3a (FOXO3a) protein, leading to an increase in the expression of antioxidant enzymes [64,65].

In conditions associated with age and inflammation, NAD^+^ plays a role in regulating the immune function of macrophages. Age-related chronic inflammation is linked to a decrease in NAD^+^ levels in aging macrophages. Accumulating evidence suggests that increasing NAD^+^ levels may have anti-inflammatory benefits in various disease models, including those associated with neurodegenerative conditions [44,64]. Finally, increased NAD^+^ availability promotes proteostasis by enhancing lysosomal and proteasomal pathways involved in protein and organelle quality control [66].

### 2.6. Clinical Insights into NAD^+^ Restoration and Metabolic Control

Precursors of NAD^+^ slow the process of aging and protect against a wide range of age-related illnesses in cellular and animal models, leading investigators to question whether their benefits can be transferred to humans. Studies on the safety of certain NAD^+^ precursors in preclinical and clinical settings, including mice, rats, and humans, have been conducted or are currently ongoing and generally demonstrate favorable safety and tolerability profiles for compounds such as NR, NMN, NAM, and NA when administered at the doses and durations studied [8,67]. A summary of the findings from studies aimed at addressing NAD^+^ levels is shown in Table 2. Interestingly, under pathological conditions, impaired conversion of NMN to NAD^+^ due to reduced NMNAT2 activity can lead to intracellular NMN accumulation, increasing the NMN/NAD^+^ ratio and activating SARM1. Activated SARM1 depletes NAD^+^, resulting in metabolic failure and axonal degeneration. Conversely, normal NMNAT2 activity maintains NAD^+^ homeostasis and protects neurons from SARM1 activation [68,69]. NR supplementation appears safe in short-term studies and increases NAD^+^ levels, but metabolic benefits seen in preclinical models have not consistently translated to humans. Therefore, redox-modulating therapies require careful evaluation of long-term safety, tissue-specific effects, and context-dependent outcomes [70].

## 3. The NADPH-Glutathione Axis in Age-Related Oxidative Stress

NADPH is a crucial electron donor and coenzyme that accepts and transfers the regenerative potential for a variety of anabolic reactions. NADPH plays a pivotal role in maintaining the redox status of cells and supplying reducing equivalents to synthetic metabolic pathways. NADP^+^/NADPH is the predominant redox couple that regulates the oxidative processes associated with cellular aging. The equilibrium between NAD^+^ and NADPH is maintained by transhydrogenase enzymes that reversibly oxidize NADPH and thereby reduce NAD^+^ and by NADK enzymes that facilitate the conversion from NAD^+^ to NADP^+^ [106].

NADPH is produced by several enzymes, including enzymes in the pentose phosphate pathway, and the NADP-dependent isocitrate dehydrogenases (NADP-IDHs). Certain reactive molecules and by-products of free radical-induced oxidation, such as nitric oxide and 4-hydroxynonenal, can react with the sulfhydryl groups of these NADP-IDH enzymes, leading to chemical and structural alterations and resulting in enzyme inactivation [107]. A decrease in the activity of these NADPH-synthesizing enzymes is linked to the development of neurodegenerative disorders.

The response to oxidative stress is mediated by the glutathione reductase (GR)/glutathione peroxidase (GPx) antioxidant system, which requires NADPH to neutralize reactive oxygen species (ROS). On induction of oxidative conditions by ischemia, the NADP-IDH enzymes undergo a regulatory shift that reduces their susceptibility to inhibition by Fe^2+^, H_2_O_2_, and 2-oxoglutarate, perhaps as a means of ensuring the continued generation of NADPH [108]. NADPH levels are also regulated by NADP-dependent malate dehydrogenase (NADP-MDH) in response to Fe^2+^, Ca^2+^, H_2_O_2_, and other compounds. In contrast to their effects on NADP-MDH derived from the hearts of healthy rats, NADP-MDH isolated from the hearts of ischemic rats is activated by reduced glutathione (GSH) and only slightly inhibited by oxidized glutathione (GSSG). Such alterations in the characteristics of NADP-MDH further underscore its significant contribution to the generation of NADPH for the GR/GPx antioxidant system [109].

Although NADP-dependent isocitrate dehydrogenase (NADP-IDH) and NADP-dependent malic enzyme (NADP-MDH) contribute to cytosolic NADPH production, the pentose phosphate pathway represents a major source of NADPH for maintaining cellular antioxidant capacity. The rate-limiting enzyme of this pathway, glucose-6-phosphate dehydrogenase (G6PDH), catalyzes the oxidation of glucose-6-phosphate (G6P), producing NADPH and 6-phosphogluconolactone (6PGL). 6PGL is subsequently converted by 6-phosphogluconolactonase (PGLS) to 6-phosphogluconate (6PG), which serves as a substrate for 6-phosphogluconate dehydrogenase, another NADPH-producing enzyme of the oxidative pentose phosphate pathway [110]. Under conditions of oxidative stress, G6PDH is activated by GSH and becomes less susceptible to inhibition by H_2_O_2_ and GSSG. This alteration in G6PDH regulation may facilitate its provision of NADPH for the formation of glutathione and its activation in pathological states [111]. In particular, G6PDH protects against neurodegeneration in aged mice by inhibiting oxidative DNA damage by endogenous ROS in various brain areas [112]. Overexpression of G6PDH in mice significantly reduces neuronal damage after ischemia/reperfusion injury by maintaining the activity of the antioxidant defense system [113].

NADPH levels are also maintained by mitochondrial NAD(P) transhydrogenase, which regulates mitochondrial metabolism by altering the cellular balance of NAD^+^ and NADPH. NADPH provides reducing equivalents for the regeneration of reduced glutathione (GSH) from GSSG and reduced thioredoxin, whereas the protective effects of NAD^+^ are linked, in part, to its role as a substrate for the mitochondrial sirtuin SIRT3 [114]. Under conditions of increased demand for cytosolic NADPH, mitochondrial NADPH may act as a source of reducing equivalents in the reverse NADP-IDH reaction. The isocitrate and subsequent citrate produced in this process are transported into the cytosol via the tricarboxylate transport system and are subsequently metabolized by cytosolic NADP-IDH to generate NADPH [115].

NADPH serves as an electron donor for the multi-subunit NADPH oxidase (NOX) complexes. Membrane-bound NOX enzymes transfer electrons from NADPH across the membrane to molecular oxygen, generating superoxide anions, which can subsequently form other reactive oxygen species, including hydrogen peroxide and, under certain conditions, hydroxyl radicals. NOX catalytic subunits generally contain six transmembrane domains and cytosolic dehydrogenase domains that bind FAD and NADPH. NOX enzymes participate in diverse biological processes, including immune defense, redox signaling, cellular metabolism, and regulation of gene expression [116].

NOX plays a role in neurodegenerative processes and has been identified as a potential target for their treatment. In an animal model of Parkinson’s disease (PD), increased NOX activity has been observed in the substantia nigra, a region of the brain that plays a critical role in motor function. Mild cognitive impairment and Alzheimer’s disease (AD) are associated with increased NOX activity. Proteins associated with neurodegenerative conditions such as amyloid beta (Aβ) neurodegeneration activate NADPH oxidase enzymes in microglial cells, leading to increased production of ROS and subsequent oxidative stress. This process also results in increased consumption, and therefore decreased levels, of NADPH [116]. Under conditions of oxidative stress, NADPH exerts an anti-inflammatory effect by inhibition of ROS-mediated signaling. These antioxidant effects occur primarily via three mechanisms: regeneration of reduced GSH by glutathione reductase (GSR), regeneration of thioredoxin (Trx) by Trx reductase, and prevention of self-inactivation of catalase, which breaks down H_2_O_2_ [117].

The thiol tripeptide GSH is widely distributed throughout the body and is found at high concentrations (1–2 mM) in the brain. GSH can function independently or in combination with enzymes. A decrease in GSH levels leads to increased oxidative stress in the entire cell and in specific organelles, particularly mitochondria. This results in increased lipid peroxidation and increased levels of intracellular calcium. The GSH/GSSG ratio decreases as pathological changes occur in the brain, as determined by nuclear magnetic resonance spectroscopy. For example, in AD the GSH/GSSG ratio begins to decline even before the formation of amyloid plaques or signs of neurodegeneration. Interestingly, congenital mutations in genes that encode enzymes involved in GSH biosynthesis are exceedingly rare, whereas acquired disorders of GSH metabolism are typical of neurodegenerative conditions [118]. For example, neurodegenerative processes are associated with disruption of GSH-dependent glutaredoxin systems involved in glutathionylation and deglutathionylation of proteins. Trx, which plays an important role in maintaining cellular protein thiol homeostasis, is also dependent on GSH levels [119,120]. The data from the literature on alterations in NADPH and GSH concentrations during aging and neurodegenerative processes are shown in Table 3.

In humans and experimental animal models, decreases in NAD^+^ levels due to age or development of PD results in reduced synthesis of proteins involved in the mitochondrial electron transport chain and an accompanying decrease in mitochondrial membrane potential. Reduced NAD^+^ availability can impair NADPH production by limiting the conversion of NAD^+^ to NADP^+^ and reducing the capacity of NADPH-generating pathways. Reduced NADPH availability impairs antioxidant activity by limiting the regeneration of reduced glutathione (GSH), resulting in increased oxidative stress in both cytosolic and mitochondrial compartments. This redox imbalance promotes oxidative modification of cellular macromolecules and contributes to mitochondrial dysfunction. In Parkinson’s disease, depletion of GSH has been associated with impaired mitochondrial complex I activity, increased vulnerability of dopaminergic neurons, and progressive dopamine depletion [106]. NAC is a cysteine precursor that supports glutathione (GSH) synthesis and cellular antioxidant capacity. NAC is generally well tolerated, including at higher doses used in chronic respiratory diseases; however, its therapeutic effects may vary depending on the disease, dose, and treatment duration. Therefore, long-term safety and tissue-specific effects of redox-modulating therapies require careful evaluation [144].

A spontaneous deletion of the nicotinamide nucleotide transhydrogenase (Nnt) gene in C57BL/6J mice reduces mitochondrial NADPH production and causes redox abnormalities, highlighting the importance of NADPH-dependent antioxidant and metabolic pathways in neuronal function [145]. In addition to its role in providing reduced equivalents for antioxidant activity, NADPH is involved in the regulation of other pathways that are involved in neuroinflammation, which is frequently associated with dysregulated lipid homeostasis. NADPH blocks the neuroinflammatory response mediated by microglia by inhibiting the p38 mitogen-activated protein kinase (MAPK) signaling pathway. NAPDH inhibits the translocation of the key regulator of inflammatory gene expression nuclear factor-κB (NF-κB) by affecting the production of the phospho-inhibitor of nuclear factor kappa-B kinase subunit α (p-Iκκa) and the levels of the κBα inhibitor (IκBa) [106,146]. NADPH negatively regulates the purinergic P2X7 receptor, reducing the assembly of the NLRP3-mediated inflammasome that activates the inflammatory response. Activation of the P2X7 receptor subtype serves as a signal to initiate the assembly of the NLRP3 inflammasome complex, which then activates caspase-1, leading to the maturation and release of pro-inflammatory cytokines such as interleukin (IL)-1β and IL-18 [106].

Although the NADPH-glutathione axis is widely recognized as a key regulator of redox homeostasis during aging, conflicting findings remain across different tissues and experimental models. Moreover, most studies are correlative, and the cell-type-specific mechanisms regulating NADPH and glutathione metabolism during aging remain poorly understood. Addressing these gaps will be essential for developing effective redox-based therapies.

The literature is therefore clear about the essential nature of NADPH and the antioxidant system in the prevention of neuroinflammation and neurodegeneration. One proposed means of prevention for PD and other neurodegenerative diseases and of slowing the effects of aging is the application of exogenous NADPH or GSH to the brain. However, one challenge is to select the most appropriate route of administration that will penetrate the blood–brain barrier (BBB) [147,148]. The main findings from the literature regarding the correction of NADPH and GSH levels in relation to aging and neurodegenerative conditions are shown in Table 4.

## 4. Conclusions

Disruption of the redox balance of cells during the aging process and neurodegenerative conditions leads to the development of oxidative stress, activation of neuroinflammatory responses, and apoptosis. Disrupting the redox balance can also impair autophagy, DNA repair, and protein glutathionylation. The key molecules involved in the regulation of multiple redox-dependent cytoprotective signaling pathways include NADH, NADPH, and GSH. It is logical to assume that replenishing the levels of these molecules may be an effective therapeutic strategy, as their concentrations decrease with aging and in neurodegenerative conditions, leading to a disruption of the balance between their oxidized and reduced forms. However, the involvement of these compounds in various cellular processes places certain limitations on their potential use as therapeutic agents. These compounds and their precursors, on the one hand, can improve the functioning of neurons in pathological conditions and, on the other hand, have a negative impact in some cases. For example, they may contribute to the development of neuroinflammation. Additionally, the issue of targeted delivery of these compounds to the brain remains relevant. Future therapies should focus not only on restoring redox molecule levels but also on the precise regulation of redox signaling. Understanding the cell-specific effects of NADH, NADPH, and GSH will be important for developing safe interventions for neurodegenerative disorders. Therefore, in the future, research efforts will need to focus on understanding the effects of NADH, NADPH, and GSH at different stages of a pathological process and achieving an effective concentration of the compounds in the targeted brain region or cellular compartments.

## Figures and Tables

**Table 1:** Changes in NAD^+^ Levels with Aging and Brain Pathology.

Animal Studies	*In Vitro* Studies
A decrease in NAD^+^ levels with age has been associated with impaired SIRT1 activity, decreased regulation of TFAM and ATP levels, and increased PARP and CD38 activity [39,48,49,50,51,52].	Reduced NAD^+^ levels in human neurons expressing the leucine-rich repeat kinase 2 (LRRK2) glycine-to-serine mutation (G2019S) are associated with Parkinson’s disease (PD) [53].
Drosophila with loss-of-function mutations in *Pink1* or *Parkin* exhibit disrupted NAD^+^ metabolism, including reduced levels of NAD^+^ and its precursors, nicotinamide riboside (NR) and nicotinamide mononucleotide (NMN) [54,55].	Reduction in the NAD^+^/NADH ratio in the cytoplasm of human GBA mutant neurons [56].
NAD^+^ depletion in the brain has been observed in models of familial AD, which is associated with metabolic dysfunction [57,58].	A decrease in the NAD^+^/NADH ratio in cells undergoing aging is associated with mitochondrial dysfunction [59].
The loss of NMNAT2 during typical Wallerian degeneration, which is caused by trauma, results in an increase in the ratio of NMN to NAD^+^ [60,61].	Reduction in NAD^+^ levels occurs in cholesterol-induced senescent macrophages [62].

**Table 2:** Studies Demonstrating the Correction of NAD^+^ Levels.

The Effect of NAD Level Correction	Ref	The Effect of NAD Level Correction	Ref
Animal Studies: NAD^+^ Replenishment with NR.			
NR administration was accompanied by a reduction in DNA damage, as indicated by a decrease in overall PARylation and the number of γ-H2AX foci in wild-type mice	[71]	NR supplementation ameliorated mitochondrial dysfunction in ATM-deficient *C. elegans* by activating mitophagy.	[72]
NR slowed down the decline in cognitive function in mice with AD model, which was attributed to a reduction in the levels of amyloid beta in the cortex and hippocampus.	[73]	NR alleviated disease-related symptoms in *C. elegans* by overexpressing α-synuclein, and in mice using a model of Parkinsonism with proteasome inhibition.	[74]
NR reduced motor dysfunction, extended survival time, and reduced neurodegeneration in a zebrafish model of 1-methyl-4-phenyl-1,2,3,6-tetrahydropyridine-induced dopaminergic neuron loss.	[75]	NR caused positive effects in AD models, including an increase in mitochondrial proteostasis and mitochondrial gene expression. This leads to a decrease in amyloid-β proteotoxicity and β-secretase levels. Additionally, it reduces neuroinflammation and cellular aging. Furthermore, the restoration of cognitive functions is observed	[73,76]
NR reduced tau dysregulation and neuroinflammation, as well as improved synaptic function and cognitive performance in the AD model with DNA repair deficiency	[77]	NR increased NAD^+^ levels in the brain were associated with improved cognitive, memory, and motor functions in 3xTg-ADPol^+^/^−^ mice. Restored synaptic plasticity in the hippocampus, and reduced neuroinflammation and tau phosphorylation.	[58]
NR increased the level of NAD^+^ in several cell lines, both in brain tissues and in the mitochondria of the brain, in the HD model. This was accompanied by activation of SIRT1, SIRT3, and PGC-1α, which enhances oxidative metabolism and provides a neuroprotective effect overall.	[18]	Intracortical administration of NR reduces brain damage caused by NMDA. NR protected mice from temporary and permanent hearing loss and spiral ganglion cell degeneration caused by noise exposure.	[78] [79]
Animal Studies: NAD^+^ supplementation.			
NAD^+^ improves the DNA repair capacity of neurons by deacetylating the Ku70 DNA repair protein, and restores mitochondrial homeostasis in *C. elegans*.	[63]	NAD^+^ replenishment promotes mitochondrial biogenesis via the NAD^+^/Sirt1-PGC1α pathway in aging *Caenorhabditis elegans* and murine models.	[48,80]
NAD^+^ administration has been shown to protect against methylmercury-induced dopaminergic neurodegeneration and the subsequent behavioral deficits in *C. elegans*.	[81]	NAD^+^ supplementation reduced neuroinflammation in mice subjected to sleep deprivation.	[82]
An increase in NAD^+^ levels via NMN supplementation in the diet reduces neurotoxicity and delays the progression of motor dysfunction in mice that overexpress the mutant Sod1 gene.	[83]	The increased NAD^+^ levels in the cochlea prevented cisplatin-induced tissue damage in mice by inhibiting oxidative stress, DNA damage, and inflammation.	[84,85]
The replenishment of NAD^+^ has led to an increase in lifespan and improved health in a model system of worms and mice with ataxia telangiectasia, through the support of mitophagy and DNA repair.	[72]	Exogenous administration of NAD^+^ demonstrated a neuroprotective effect against ischemic damage to neurons.	[86]
NAD^+^ therapy significantly reduced damage caused by chronic cerebral hypoperfusion in neurons of the cerebral cortex and hippocampal CA1 region, and improved cognitive deficits.	[87]	NAD^+^ treatment has been shown to protect against dopaminergic neurodegeneration and behavioral impairments in a model of PD in *C. elegans*.	[88]
Animal Studies: NAM Consumption.			
NAM protects against amyloid-β-induced oxidative damage and mitochondrial dysfunction in synaptic structures that are critical for memory and learning in rats	[89]	NAM inhibits neurodegeneration in flies with mutant Parkin NAM restores cognitive deficits in AD models by modulating tau phosphorylation and attenuating the deficits in spine density in the hippocampal primary neurons. NAM restores cognitive deficits in AD models by modulating tau phosphorylation and attenuating the deficits in spine density in the hippocampal primary neurons.	[54] [90,91]
NAM supplements support mitochondrial integrity, reduce the levels of pathological plaques and hyperphosphorylated tau, and maintain microtubule stability in an AD model.	[88,90]	NAM Enhanced Mitochondrial Function and Protected Against Locomotor Deficits in the Drosophila PD Model.	[92]
*In vitro* Studies: NAD^+^ replenishment with NR.			
NR may prolong the replicative lifespan of wild-type yeast. NR administration resulted in an increase in the levels of NAD^+^ and NAM, an increase in the number of mitochondria, and an increase in TFAM. This contributed to the restoration of mitochondrial function in an iPSC neural cell model derived from PD patients with 3 different genetic mutations (N370S, L444P, RecNci)	[93] [56]	NR enhanced the efficiency of DNA repair and decreased the number of micronuclei in cells exposed to X-rays, as well as in Atm-deficient mouse neurons.	[72,94]
*In vitro* Studies: NAM consumption.			
NAM reduces the risk of glaucoma in D2 retinal ganglion cells and inhibits mitochondrial loss and fragmentation in retinal ganglion cells during complex I inhibition caused by rotenone	[95,96]	NAM significantly enhances DNA repair in gamma-irradiated XP cells *in vitro*	[97]
Clinical studies: NAD^+^ supplementation.			
Administration of NADH, twice daily at a dosage of 10 mg per day, for 8 to 12 weeks, has been shown to improve cognitive function in patients with AD.	[8]	NADH (intravenous administration, 25 mg/day) for 4 days resulted in significant improvement or moderate improvement in motor deficits in PD patients.	[98]
NADH therapy was accompanied by positive clinical outcomes in PD patients, as well as an increase in plasma levels of levodopa.	[44]	In elderly volunteers, an increase in NAD levels in extracellular vesicles enriched with neurons (NEV) was accompanied by a reduction in amyloid-beta 42 (Aβ42) levels and neuroinflammatory markers.	[99]
NADH treatment has been shown to slow the progression of dementia, improve speech fluency, and enhance visual-constructive abilities among patients.	[88]	Intravenous administration of NADH has been shown to improve motor symptoms in patients with PD.	[88]
Clinical studies: NA supplementation.			
500 mg of NA administered twice daily resulted in reduced rigidity and bradykinesia in a 78-year-old patient with PD	[100]	Niacin improved motor and cognitive function in patients with PD. Acipimox, a NA analog, enhances the mitochondrial function of skeletal muscle in patients with type 2 diabetes mellitus	[44] [101]
Clinical studies: NR supplementation.			
NR improves mitochondrial function in neurons derived from human induced pluripotent stem cells with PD and restores the phenotype in Drosophila models with the same condition [56].	[56]	Administration of NR led to a reduction in the level of pro-inflammatory cytokines in cerebrospinal fluid and alterations in the expression of genes associated with mitochondrial function, antioxidative activity, and protein degradation pathways in peripheral tissues of PD patients.	[102]
In healthy volunteers, taking NR increases the level of NAD^+^ in the brain.	[103]	Treatment with NR has resulted in a clinical reduction in ataxia and an improvement in eye movements in patients with AT.	[104,105]

**Table 3:** Changes in NADPH and GSH Levels during Neurodegeneration and Aging.

Animal Studies	Clinical Studies
NAD(P)H and GSH depletion were linearly correlated with neuronal death in aged mice with AD [121].	Patients with AD were found to have a decrease in the levels of GSH and the GSH/GSSG ratio in the brain, compared to healthy individuals [122,123].
Excessive activity of G6PD and increased formation of NADPH in microglia, contributed to the activation of NOX2, resulting in the production of excessive amounts of ROS in a chronic PD model in mice [124].	Patients with mild cognitive impairment showed a decrease in the GSH/GSSG ratio [125].
A decrease in the concentrations of NADPH and GSH has been observed in aging rats [126].	Postmortem examination of brain tissues from normal individuals with the presence of random Lewy bodies and the loss of nerve cells in the substantia nigra revealed a decrease in the levels of GSH within the substantia nigra [127].
The knockout of the catalytic subunit of GCLC in mice leads to brain atrophy, accompanied by a loss of neurons and neuroinflammatory processes [128].	A decrease in the concentration of GSH in the substantia nigra has been observed in patients with progressive supranuclear palsy [129]. A reduction in GSH levels was noted in the blood plasma of HD patients [130].
A decrease in the concentration of GSH in the spinal cord has been observed in a mouse model of ALS [131].	Loss of ATM function leads to a reduction in G6PD levels in patients with ataxia [132].
A reduction in GSH levels was observed with age in multiple tissuesof aging rodents [133].	Clinical studies using nuclear magnetic resonance spectroscopy have shown a decrease in GSH levels in multiple sclerosis patients [134,135].
***In vitro* studies**	
Inhibition of intracellular GSH synthesis leads to neuroinflammation in human microglial and astrocytic cells [136].	The concentration of GSH in the hippocampal region of postmortem human brains decreases with age [137].
Soluble amyloid-β oligomers inhibited the EAAC1-mediated cysteine uptake process, which resulted in a decrease in the levels of GSH in cultured human neurons [138].	A higher baseline level of GSH in blood plasma has been associated with a lower risk of developing asthma and improved long-term preservation of cognitive functions [139]. A reduction in GSH levels was noted during post-mortem examination of the substantia nigra in the brains of PD patients [140].
The expression of the mutant form of A53T α-synuclein in cultures of dopaminergic neurons resulted in depletion of GSH and mitochondrial dysfunction [141].	Patients with ALS are characterized by reduced levels of G6PD, accompanied by decreased NADPH levels, reduced levels of GSH, and reduced activity of Gsr and G6PDH in red blood cells. Studies using 1H-MRS have demonstrated a reduction in GSH levels in motor cortex [142,143].

**Table 4:** Studies Demonstrating the Correction of NADPH and GSH Levels.

The Method of NADPH and GSH Level Correction	The Effect of NADPH and GSH Level Correction
Animal and *in vitro* studies	
NADPH administration	NADPH has the potential to reduce the neuroinflammatory response in a murine model of Parkinson’s disease induced by MPTP [149].
	NADPH has a neuroprotective effect, which is achieved by increasing the levels of GSH and reducing the levels of ROS in the midbrain of mice. This effect is achieved through the inhibition of the activation of glial cells, the translocation of nuclear factor κB (NF-κB), and the phosphorylation of p38 mitogen-activated protein kinase (MAPK) in PD caused by MPTP [149,150].
	NADPH application significantly alleviated NLRP3 inflammasome activation in microglia and exerted neuroprotective effects via suppression of ATP-induced P2X7R activation [151].
	After administration of NADPH and N-acetylcysteine (NAC) to retinal ganglion cells in rats with chronic ocular hypertension and cells subjected to oxygen-glucose deprivation, apoptosis, axonal damage, and peroxidation were reduced [152].
	The combined administration of NADPH and NAD^+^ produces a more potent neuroprotective effect compared to using both compounds individually in the treatment of ischemic stroke [153].
Addition of citric and malic acid, dieckol, and resveratrol	The addition of citric and malic acids increased the levels of NADPH, while small molecules such as dieckol and resveratrolincreased the expression of IDH1 and ME1, in G6PD-deficient microglial cells. This combined approach restored redox homeostasis and lysosomal function [154].
NAC administration	These findings suggest that N-acetylcysteine (NAC), in combination with artemisinin, resveratrol, and hesperidin, exerts multi-target neuroprotective effects in an experimental AD model, potentially through modulation of oxidative stress, neuroinflammation, and apoptotic pathways [155].
	NAC increased the levels of enzymatic and non-enzymatic antioxidant activity, while significantly decreasing the levels of prooxidant activity and markers of inflammation, in aged rats [156].
Clinical Studies	
NAC supplementation	Intravenous administration of NAC has been shown to increase GSH levels in PD patients [157].
	Oral administration of NAC resulted in an increase in peripheral blood antioxidant levels in patients with PD, but no significant increase was observed in brain GSH levels [158].
	Nutritional supplementation containing NAC, together with other bioactive compounds, has been shown to improve cognitive function in patients with AD [159,160].
NADPH supplementation	NADPH treatment was associated with improved clinical disability in a small group of patients with Parkinson’s disease and increased urinary HVA levels, indicating stimulation of endogenous L-DOPA production [161].

## Data Availability

No new data were created or analyzed in this study. Data sharing is not applicable to this article.

## Abbreviations

GSSG	oxidized glutathione
GSH	reduced glutathione
ROS	reactive oxygen species
NAD^+^	Nicotinamide adenine dinucleotide (oxidized form)
NADH	Nicotinamide adenine dinucleotide (reduced form)
NADP^+^	Nicotinamide adenine dinucleotide phosphate (oxidized form)
NADPH	Nicotinamide adenine dinucleotide phosphate (reduced form)
Gr	glutathione reductase
GPx	glutathione peroxidase
NMN	Nicotinamide mononucleotide
FADH_2_	Reduced flavin adenine dinucleotide
TCA cycle	Tricarboxylic acid cycle
NAM	Nicotinamide
NAMN	Nicotinic acid mononucleotide
NR	Nicotinamide riboside
PARP1	Poly(ADP-ribose) polymerase 1
Trp	Tryptophan
ATP	Adenosine triphosphate
CD38	Cluster of differentiation 38
SIRT	Sirtuin
